# Development of subgingival calculus detector utilizing optical fiber: Verification of its potential for clinical application

**DOI:** 10.1371/journal.pone.0314563

**Published:** 2024-12-03

**Authors:** Tsubasa Kato, Kotaro Sena, Risa Ishiko, Naoko Tanda, Nobuhiro Yoda, Hiroki Hihara, Takeyoshi Koseki

**Affiliations:** 1 Department of Oral Supportive Care and Management, Tohoku University Hospital, Sendai, Miyagi, Japan; 2 Division of Preventive Dentistry, Department of Community Social Dentistry, Tohoku University Graduate School of Dentistry, Sendai, Miyagi, Japan; 3 Division of Advanced Prosthetic Dentistry, Department of Rehabilitation Dentistry, Tohoku University Graduate School of Dentistry, Sendai, Miyagi, Japan; Universidade Federal de Pelotas, BRAZIL

## Abstract

The removal of subgingival deposits, especially calculus, plays a crucial role in basic periodontal therapy. However, manual detection methods affect accuracy owing to the operator’s skill. To avoid this uncertainty, we have developed a calculus detection device named “Sensor probe” and evaluated its ability to detect calculus for future clinical applications. The Sensor probe consisted of a 635 nm-wavelength semiconductor laser and a 0.5 mm-diameter single-mode optical fiber. Initially, the performance of the device was evaluated using clinically obtained extracted teeth with calculus covered with a stainless-steel shielding plate with pinhole. Then, the effect of the optical fiber’s end shape on calculus detection performance was analyzed. Lastly, the performance of the Sensor probe was compared to that of a conventional periodontal probe in terms of accuracy, sensitivity, and specificity for calculus detection using calculus-covered extracted teeth. The results indicated that Sensor probe detected dental calculus through the pinhole with a diameter of 300 μm or more when applied from a distance of 100 μm. The results analyzing the effect of the optical fiber’s end shape on calculus detection performance showed that cutting the fiber end at an angle of 45° resulted in the most effective calculus detection. This may be because the laser light refracted on the cut surface and concentrated on the fiber side. Moreover, by comparing the performance of this device to a conventional periodontal probe revealed that the Sensor probe showed improved calculus detection accuracy in deeper periodontal pockets. This improvement was particularly significant in the apical third, where detection is typically difficult. In conclusion, a Sensor probe that uses an optical fiber with a 45° angled end may facilitate subgingival calculus detection. In future clinical applications, Sensor probes could lead to more accurate and efficient calculus removal, especially for deeper periodontal pockets.

## Introduction

The most basic approach for periodontitis treatment is root surface debridement, i.e., scaling and root planing [1–4]. In cases of deep periodontal pockets (≥ 6 mm), periodontal surgery with root surface debridement under direct vision should be considered. However, when surgical treatment is not applied due to the patients’ underlying general conditions, non-invasive approaches, such as repeated scaling and root planing, should be considered. However, especially in deep periodontal pockets, the results of nonsurgical treatment are highly dependent on the operator’s skill [5, 6]. Therefore, accurately detecting and removing calculi from deep periodontal pockets is a significant challenge for the successful treatment of advanced periodontitis.

The exact role of subgingival calculus in the initiation progression of periodontal disease is debatable [7]. Calculus itself does not induce inflammation but provides an ideal surface for microbial colonization [8] and it has been demonstrated that epithelial adherence to subgingival calculus can occur following its disinfection with chlorhexidine that is compatible with a clinically acceptable level of gingival wound healing [9]. Remaining calculus after periodontal treatment is considered to have a deleterious effect because of its ability to provide an ideal surface for microbial colonization. For this, the rationale for calculus detection and their removal relates to eliminating root surface irregularities harboring pathogenic bacteria. Further, recent in vitro findings indicates that the pathologic risk of calculus goes beyond the retention of biofilm and may represent a different pathophysiologic pathway for periodontal disease separate from the direct action of biofilm [10]. It was demonstrated that sterile calculus, when phagocytized by connective tissue cells and macrophages in cell culture, induced cell death that may lead to breakdown of periodontal tissue integrity [11, 12]. Taken together, detection and removal of calculus plays an important role in success of periodontal therapy.

The conventional method with periodontal probes and explorers requires operator skill, leading to significant variance in calculus detection outcomes among operators [13]. In response to this issue, alternative methods have been developed to compensate for the possible differences among operators when using conventional hand instrumentation [14, 15], including fiberoptic endoscopy [16–18], spectro-optical technology [19, 20], autofluorescence-based techniques [21–26], and ultrasound techniques [27–29]. Among these methods, the ultrasound (Perioscan^®^) or autofluorescence (Keylaser 3^®^) techniques are currently installed in calculus removal devices.

Utilizing autofluorescence-based techniques, laser-based fluorescence with 635 or 655 nm excitation was reported to be a powerful tool for subgingival calculus detection [25]. Kurihara *et al*. imaged extracted teeth with subgingival calculus and found that dentine caries emits a characteristic 700 and 720 nm emission when excited by 635 and 655 nm light, respectively. They further showed that 635 nm excitation was more capable of differentiating between subgingival calculus and dentin via differentiation ratios and imaged subgingival calculus deposits on the root surfaces of extracted single- and multi-rooted teeth [25]. Currently, however, no calculus detector uses 635 nm excitation laser light, which is the optimal wavelength most suitable for identifying calculi. Additionally, to detect the autofluorescence of the calculus, an optical fiber must be inserted into the gingival sulcus; however, the optimal optical fiber for insertion and the shapes of the end with maximal performance are yet to be determined.

Therefore, this study aimed to develop the technology for a new calculus detector by verifying the calculus detection capability of a semiconductor laser with a wavelength of 635 nm and by validating the changes in the calculus detection efficiency due to the shape of the optical fiber end. Furthermore, we compared the calculus detection accuracy of the developed calculus detection technology with that of a normal periodontal probe and discussed the possibility of clinical application of this device.

## Materials and methods

### Structure of a novel dental calculus detector

**Fig 1:**
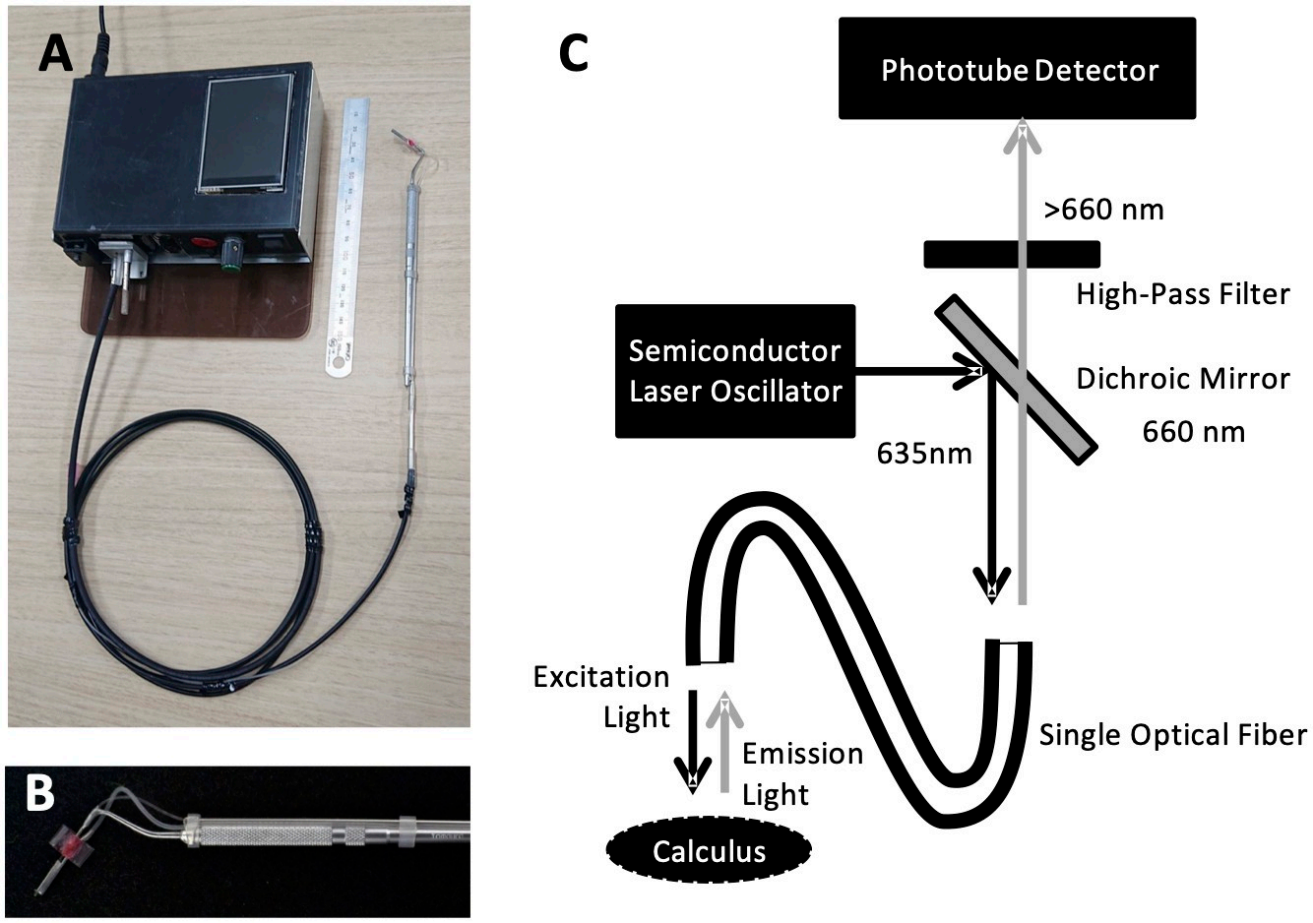
Structure of a novel dental calculus detector. (A) Calculus detection device and Sensor probe. The black housing is the main unit of the calculus detection device (Size: 14.7 x 10.0 x 4.5 cm). (B) Sensor probe. A rotation mechanism was attached to the tip of a conventional periodontal probe to change the orientation of the optical fiber end in the gingiva. (C) Principle of calculus detection by Sensor probe. The semiconductor laser oscillator emits a 635 nm-wavelength laser beam that was reflected by the dichroic mirror. The laser reaches the end of the optical fiber, where it irradiates the calculus and generates fluorescence, which returns to the device through the same fiber. The returned fluorescence passes through a dichroic mirror and a high-pass filter (cutoff value of 660 nm), which is installed for noise reduction, and finally reaches the phototube detector. The resulting electrical signal output from the phototube detector was used as the fluorescence intensity of calculus (FIC). FIC was adjusted to the value at which the fluorescence intensity of the standard fluorescent plate was 100.

The main unit of the developed calculus detector consists of a semiconductor laser oscillator, light receiver, and controller (Fig 1A). An optical fiber (diameter 0.5 mm; single mode) was connected to this main unit, and the end was fixed to the tip of the periodontal probe, hereinafter referred to as “Sensor probe” (Fig 1B). Fig 1C shows the calculus detection principle of this device. A sounder and touch monitor attached to the main unit detected the calculus using sound and visual graphics.

### Evaluation of calculus detection ability

The study received ethical approvals from the Research Ethics Committee of Tohoku University Graduate School of Dentistry (approval numbers: 19–24 and 2019-3-5). The teeth extracted due to severe periodontal disease with calculus were collected between 14/05/2008 and 30/05/2020. All patients provided written informed consent for the use of their teeth. The teeth with subgingival calculus were cleaned by removing as much plaque and soft tissue as possible with a toothbrush and then stored at 4°C in a saline solution. All patient data were fully anonymized before processing the teeth for research.

**Fig 2:**
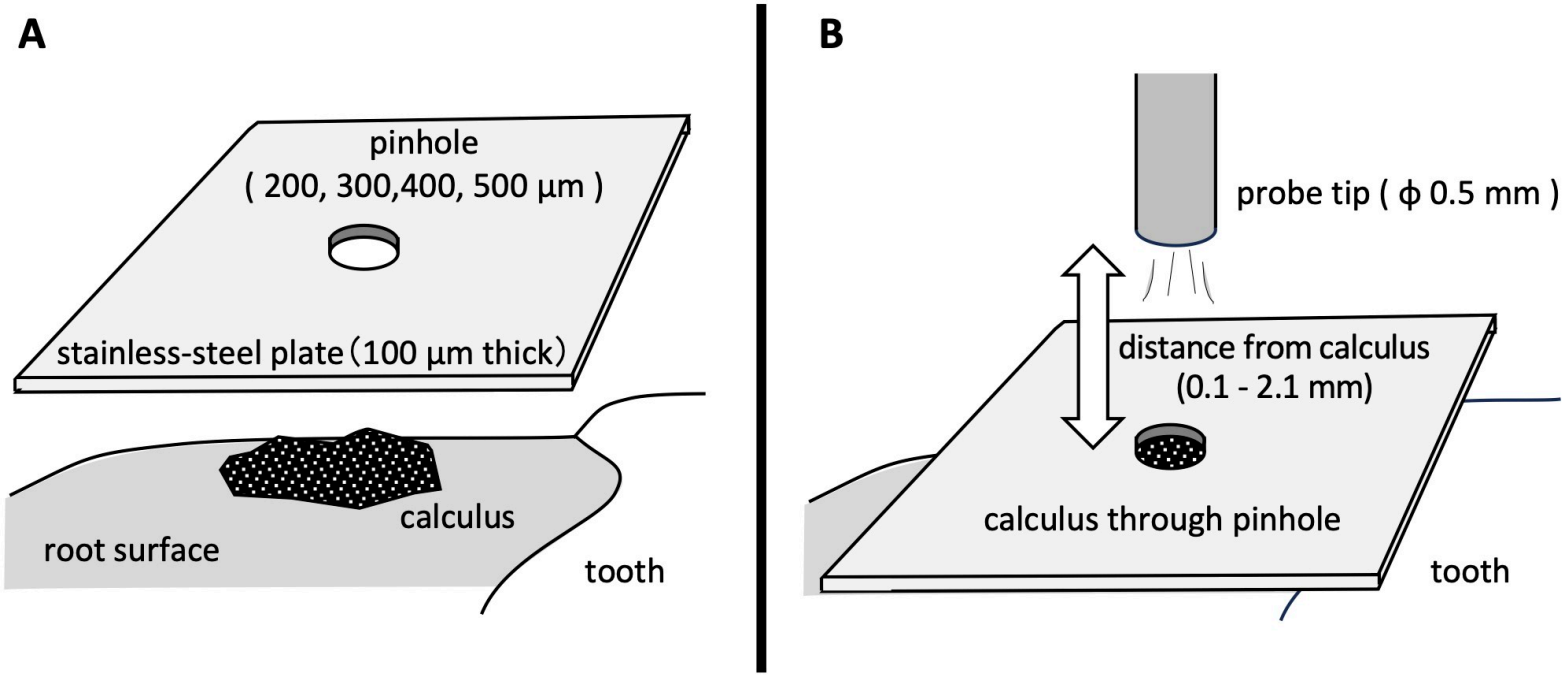
Schematic diagram of measuring fluorescence intensity of calculus (FIC). (A) The apparent size of the calculus changed depending on the pinhole size in the stainless-steel shielding plate placed in contact with it. (B) The effect of fiber end/calculus distance on FIC was also evaluated. The change in FIC was recorded as the distance between the optical fiber end and the calculus increased (0.1, 0.6, 1.1, 1.6, 2.1 mm) through a 400 μm diameter pinhole. The same experiment was performed without the stainless-steel shielding plate.

To evaluate the calculus-detection ability of the Sensor probe, effect of the calculus size and the distance between the optical fiber end and the calculus was analyzed. For evaluating the size of the calculus, a stainless-steel shielding plate (100 μm thick) with a pinhole to define the apparent size was used to measure FIC. The pinholes were 200, 300, 400, 500 μm in diameter (manufactured by the Technical Office, Institute of Multidisciplinary Research for Advanced Materials, Tohoku University). The stainless-steel shielding plate was placed in contact with the calculus of the extracted tooth and fixed using a modeling compound (GC Co., Tokyo, JAPAN). Next, an optical fiber cut at an angle of 90° (C-type) was placed directly above the pinhole (Fig 2A), and FIC was measured. A similar measurement of the unobstructed calculus was performed after removing the shielding plate. In order to evaluate the effect of distance from the optical fiber end, FIC was measured with C-type optical fiber and a stainless-steel shielding plate with 400 μm pinholes when the distance of the fiber end to the calculus was 0.1 mm, 0.6 mm, 1.1mm, 1.6 mm, or 2.1 mm (Fig 2B).

### Fluorescence intensity distribution of calculus on the root surface

The teeth with calculi were arranged in a straight line on an XY table with computer-controlled XYZ motion stages (SIGMAKOKI Co., Ltd., Tokyo). The optical fiber was fixed on a Z-stage perpendicular to the XY table and could move vertically. The teeth were moved forward, backward, left, and right at 333 μm intervals, with the optical fiber moving systematically up and down to make contact with the tooth during each movement to measure FIC. This motion was performed on a 10 mm square area of the tooth. Subsequently, a distribution map of FIC was generated.

### Verifying the effect of the shape of the optical fiber end on calculus detection performance

To verify the influence of the shape of the fiber end on calculus detection, the fiber end was processed into five different types: a 90° angle cut to the optical axis (C-type), a 45° angle cut to the optical axis (B-type), a flame-treated C-type forming an umbrella shape (F-type), a heater-treated C-type with a wider canopy than that of the F-type (T-type), and a polished and rounded C-type (R-type). The main unit of the Sensor probe connected each end-shaped optical fiber. The end was then placed parallel to the surface of a white screen at a distance of 1 mm. The diffused state of the laser light projected onto the white screen was recorded in a photograph. Photographs were subjected to black-and-white reverse processing using Photoshop CC 2015 (Adobe Systems Inc., San Jose, CA, USA).

The extracted teeth were fixed in line on the XY stage of the machine used in the experiment on the fluorescence intensity distribution of the calculus on the root surface. The stage was moved in the X-axis direction at 333 μm intervals, and fluorescence was measured at each location using five types of optical fiber that had different end shapes. To prevent excessive pressing of the fiber against the calculus, a buffer mechanism was installed that lifts the fiber when it comes into contact with the calculus, allowing it to press against the calculus only with its own weight (about 0.2 N). Assuming clinical practice, the experiment was performed with the fixed angle of the optical fiber changed from 10°, 20°, or 90° to the tooth surface. The above experiment was conducted for each prepared optical fiber using five different end shapes.

### Detection of calculus in deep pockets using a Sensor probe

**Fig 3:**
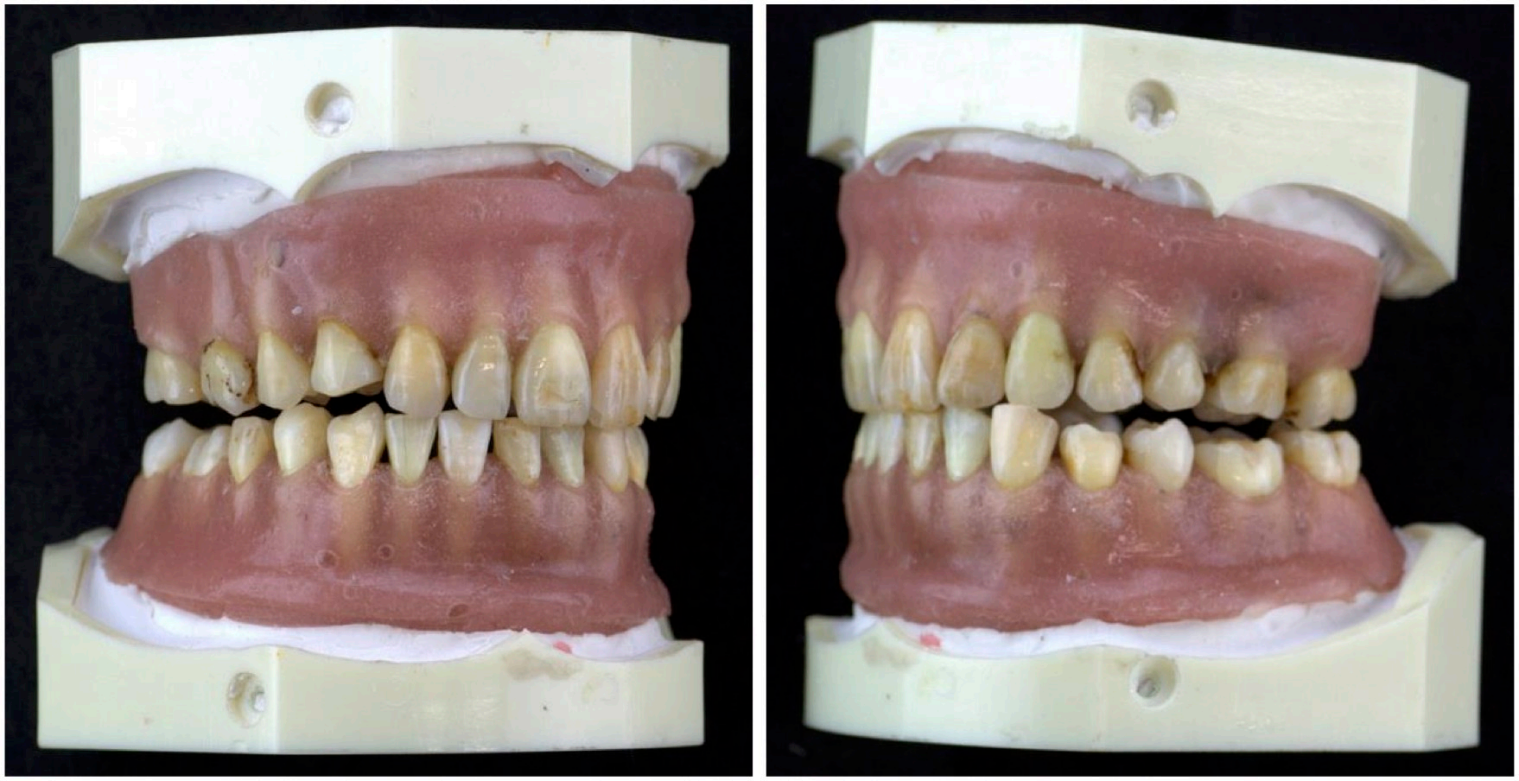
Periodontal pocket dentition model. Periodontal pocket dentition model was prepared utilizing extracted teeth with calculi and the tooth roots were covered by an artificial gingiva.

Extracted teeth with calculi that partially adhered to the root surface were selected from all types of maxillary and mandibular teeth. Dowel pins were attached to the apex of the teeth and placed on plaster pedestals in the dental arch. In addition, an artificial gingiva made of soft silicone for epithesis (GC Co., Tokyo, Japan) was used to cover the cementoenamel junction and conceal the calculus-adhering area. This periodontal pocket dentition model (Fig 3) allows probing of the entire tooth up to the apical part.

Two dentists with more than four years of clinical experience participated in the experiment as operators (Op1, Op2). After receiving instructions on the Sensor probe, each operator practiced the operation for 5 min using an extracted tooth with calculus. The periodontal pocket dentition models were mounted on the upper and lower jaws of a manikin (Nissin Dental Products Inc., Tokyo, Japan). The operators then probed the maxillary teeth using a regular periodontal probe (Williams^®^, Hu-Friedy Mfg. Co., Chicago, IL, USA) and recorded the calculus detection sites on a worksheet. The mandible was then probed with a Sensor probe, and the calculus detection sites were recorded on a worksheet. The process was repeated using a regular periodontal probe for the mandible and a Sensor probe for the maxilla at intervals of several days. The calculus detection results obtained from both probes at the same site were compared and verified based on the worksheet.

To calculate detection accuracy, calculus attachment sites were defined as those easily visible to the naked eye under bright light. The tooth roots were divided into nine sections: mesial, central, and distal on the buccal and lingual/palatal surfaces, and cervical, middle, and apical sections on the same surfaces. The molars included the bifurcation areas. The accuracy, sensitivity, and specificity of each segment were calculated by comparing the results of the calculus detection by each operator using a periodontal or Sensor probe.

The results between the Sensor probe and conventional manual probe or between the two operators were compared using the χ2 test. A *p*-value <0.05 was considered a significant difference. All statistical analyses were performed using PASW Statistics 18 software (IBM Japan, Tokyo, Japan).

## Results

### Performance of the calculus detector

**Fig 4:**
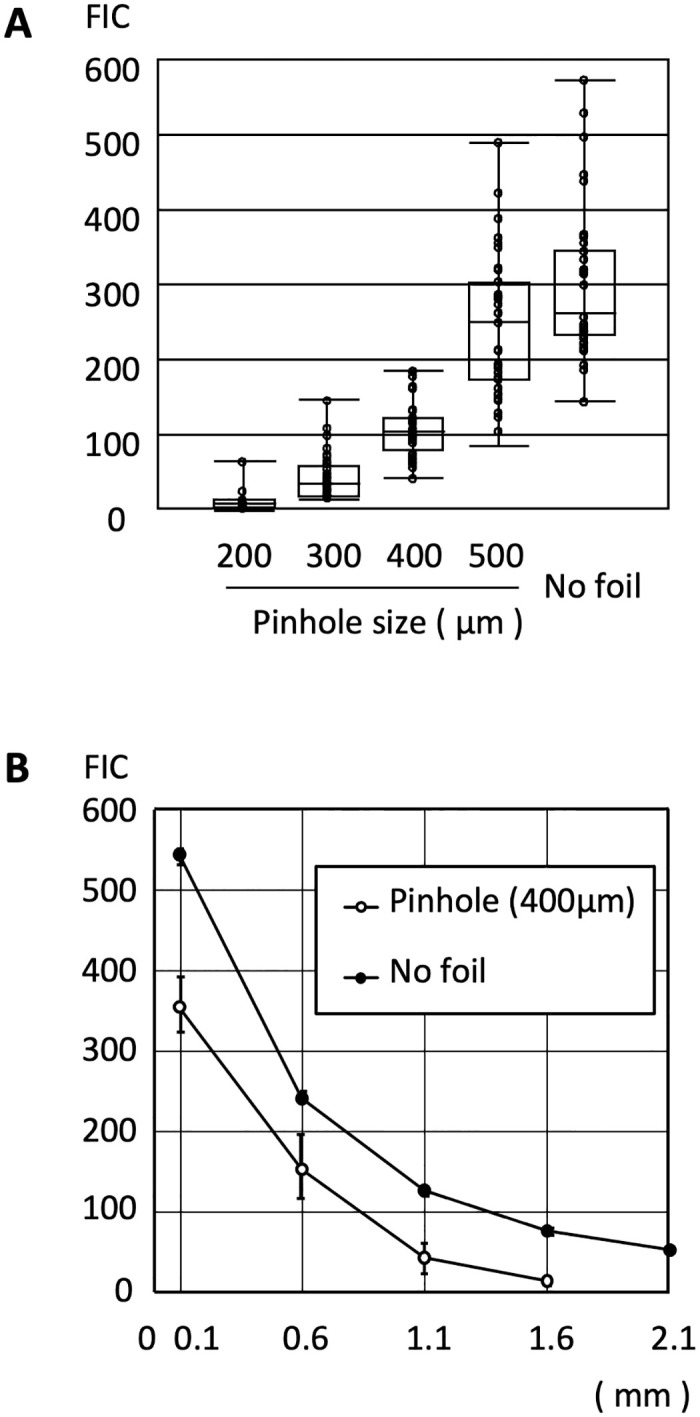
Effect of factors affecting FIC. (A) FIC of various apparent calculus sizes. FIC was measured with C-type optical fiber when a stainless-steel shielding plate with 200, 300, 400, or 500 μm pinholes was placed on the calculus and when no stainless-steel shielding plate was provided. (B) FIC depends on the distance from the optical fiber end. FIC was measured with C-type optical fiber and a stainless-steel shielding plate with 400 μm pinholes when the distance of the fiber end to the calculus was changed from 0.1 mm to 2.1 mm.

The relationship between FIC and the size of the pinhole is shown in Fig 4A.

The maximum FIC was recorded without the stainless-steel shielding plate. FIC decreased as the pinhole diameter decreased. When using a pinhole diameter of 200 μm, FIC could not be recognized when overlapping with signal noise. Therefore, calculus detection was possible only when the apparent calculus size was 300 μm or larger, 100 μm away from the end of the fiber.

Fig 4B shows FIC decreased with increasing distance between the calculus and the optical fiber end, both with and without the use of a stainless-steel shielding plate with a 400 μm diameter pinhole. Increasing the distance between the calculus and the fiber end by just 0.5 mm, from 0.1 mm to 0.6 mm, reduced FIC by half. A calculus with an apparent size of 400 μm was no longer detectable at a distance of 1.1 mm. This highlights the importance of maintaining the optical fiber end as close as possible to the calculus.

### Detection of calculus distribution on the root surface

**Fig 5:**
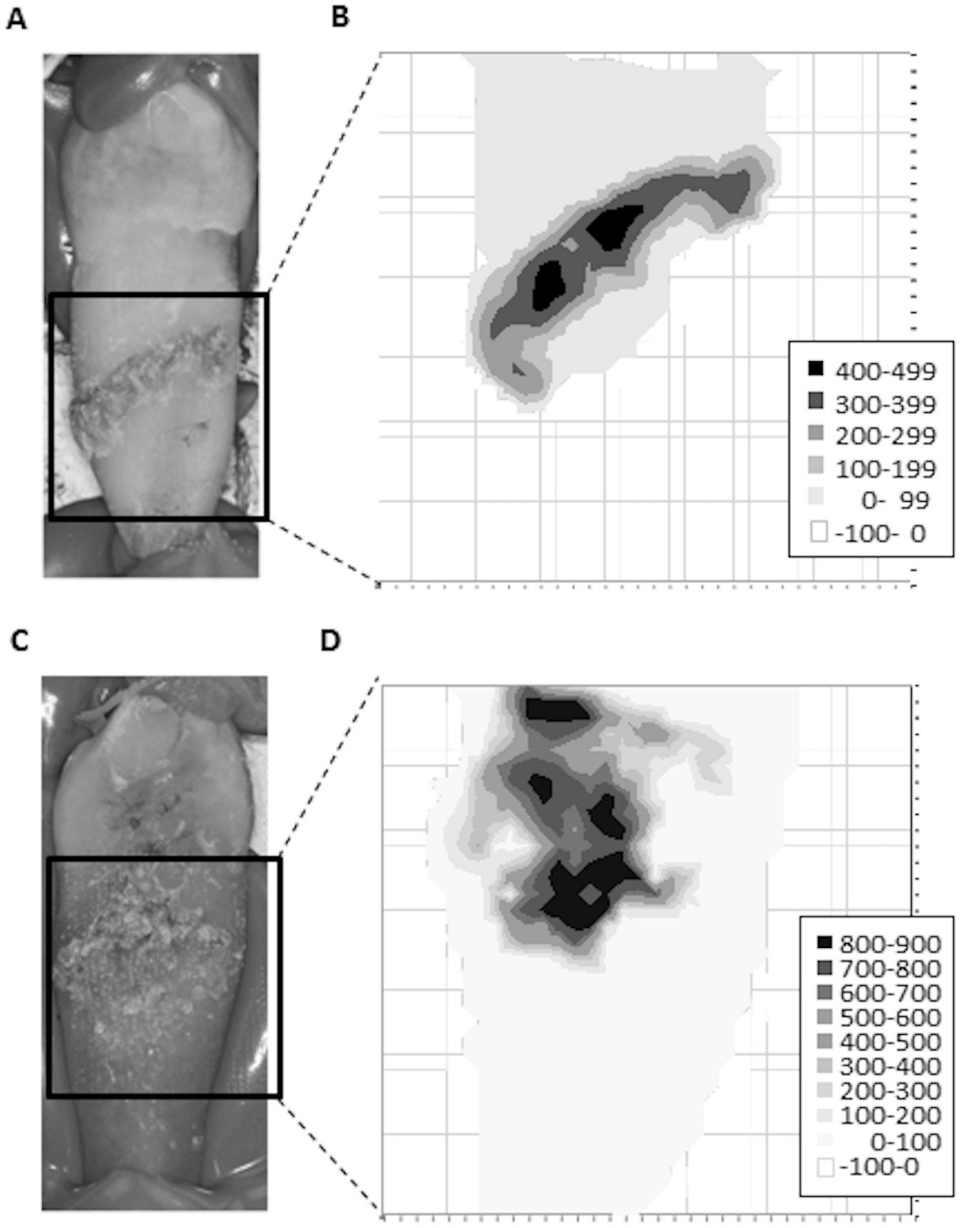
FIC distribution of root surfaces with calculus. The results of FIC measurements in the area enclosed by the bold square in tooth photographs (A) and (C) are mapped in (B) and (D), respectively.

Fig 5 shows the results of FIC measurements of the extracted teeth using a C-type optical fiber as a fluorescence distribution map. Fig 5B and 5D show the measurement results of the area (10 mm^2^) within the bold frame of the extracted tooth photograph in Fig 5A and 5C, respectively. Only the calculus adhesion sites showed strong fluorescence, while the enamel, dentin, and cementum emitted almost no fluorescence.

### Calculus detection performance with various shapes of optical fiber ends

**Fig 6:**
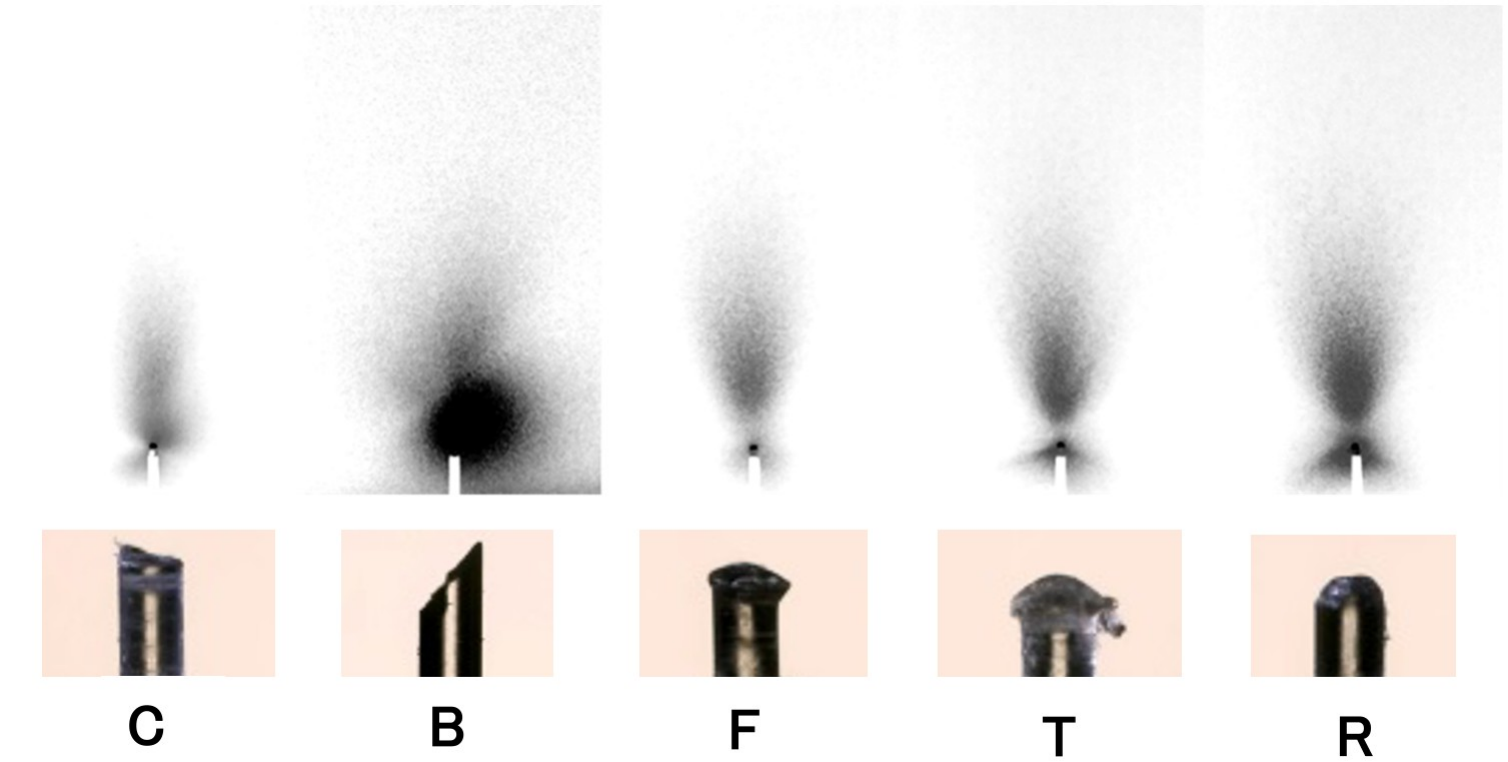
End shapes of each processed optical fiber and diffusion of laser light. The lower panels show the shape of the processed optical fiber end, which was 0.5 mm in diameter. The upper panels show the shapes formed by light projected from the optical fiber ends. C: Crosscut at an angle of 90°. B: Cut at an angle of 45° to the optical axis. F: Flame-treated C-type fiber ends forming an umbrella shape. T: Heater-treated C-type fiber ends with a wider canopy than the F-type. R: Rounded and polished end.

The diffusion of the laser light at various fiber ends displayed different characteristics, as shown in Fig 6. In the C type, the light emitted from the cut surface gently diffuses radially. The B-type fiber exhibited directional light emission with a strong regional intensity. The F- and T-type fibers exhibited light reflection from the umbrella, resulting in radial diffusion towards the fiber side. The T-type had a stronger reflected light intensity than the F-type. The R-type exhibited even stronger light reflection towards the fiber side than the T-type. Among the five prepared fiber end shapes, the B-type had the highest light emission on the side of the fiber, which was biased in a particular direction relative to the cutting plane.

**Fig 7:**
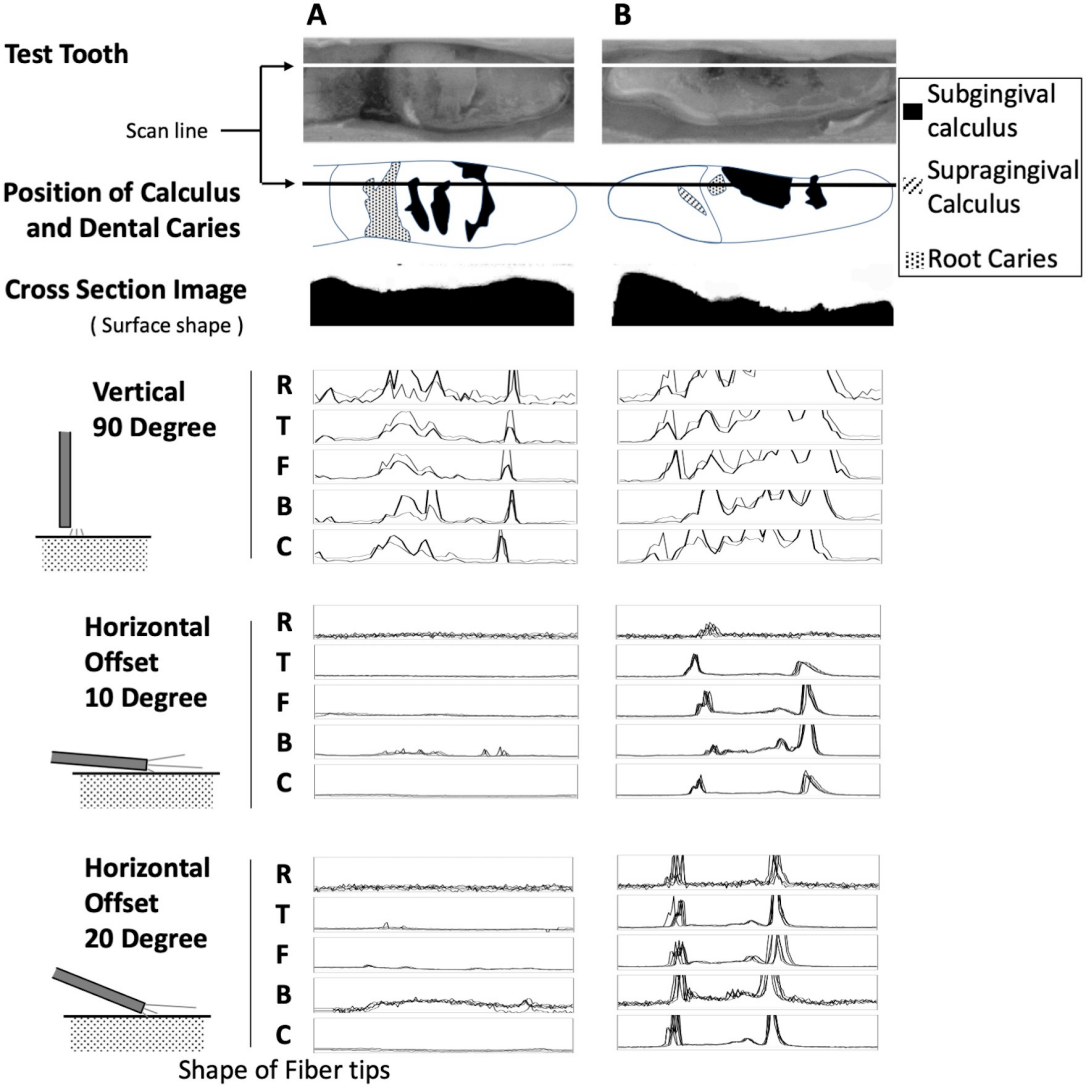
Optical fiber end shapes and FIC on the tooth surface. Each optical fiber was used to perform linear measurements of the white line area (scan line) shown in the photographs of the two extracted teeth (A) and (B). The cross-sectional images below the schematic of the root surfaces show the unevenness of the tooth surface when the scan line is viewed from the side. The graph shows the scans of FIC along the white line using fibers with various ends and placing the fibers on the tooth surface in three different directions.

Fig 7 shows the scanned FIC of the root surface of the extracted teeth when various ends of the optical fiber were placed perpendicular and at 10° and 20° to the tooth surface. In tooth A and B, when the optical fiber end was positioned perpendicular to the tooth surface (upper row), calculus was detected in all fiber end shapes. However, when the optical fiber end was positioned at an angle of 10° (middle row) or 20° (lower row) to the tooth A surface for clinical use, only the B type showed obvious FIC in the presence of calculus in tooth A, with a less uneven calculus distribution. In tooth B, which had a noticeably uneven calculus, all optical fiber end shapes could be detected, and the B-type had the greatest FIC.

Hence, when sorting the fiber ends in order of calculus detection sharpness, the ranking was B-type > F-type = T-type > C-type > R-type. The R-type has a higher noise level, leading to a poor signal-to-noise (S/N) ratio and poor performance in calculus detection.

### Detection of calculus in deep periodontal pockets

A holder was used to rotate the fiber end such that the cut surface of the B-type optical fiber maintained a constant orientation with respect to the tooth surface. Table 1 shows the results of subgingival calculus detection performed by the two dentists using a type-B fiber end on the periodontal pocket dentition mode.

**Table 1:** Detection of subgingival calculus using Sensor probe or a conventional periodontal probe.

Operator	Calculus position	N	Sensor probe			Conventional probe			*p*-value	
			Accuracy	Sensitivity	Specificity	Accuracy	Sensitivity	Specificity		
Total	Total	1128	64%	41%	77%	54%	49%	56%	<0.001	**
	Cervical	384	55%	38%	77%	56%	50%	64%	0.771	
	Middle	384	63%	46%	72%	53%	54%	52%	0.006	**
	Apical	360	75%	42%	80%	52%	33%	56%	<0.001	**
Op1	Total	564	61%	37%	74%	45%	57%	37%	<0.001	**
	Cervical	192	54%	35%	78%	55%	56%	53%	0.771	
	Middle	192	59%	35%	74%	50%	63%	42%	0.006	*
	Apical	180	69%	54%	72%	28%	46%	25%	<0.001	**
Op2	Total	564	67%	45%	80%	63%	41%	76%	0.134	
	Cervical	192	57%	41%	76%	58%	44%	75%	0.837	
	Middle	192	66%	56%	71%	55%	44%	62%	0.037	*
	Apical	180	80%	31%	88%	77%	19%	86%	0.443	

*p < 0.05,

**p < 0.01

For operator Op1, Sensor probe significantly improved the calculus detection accuracy in the middle (59% vs. 50%) as well as the apical 1/3 root surface (69% vs. 28%). For operator Op2, the performance of calculus detection in the middle of the root surface significantly improved when Sensor probe was used (66% vs. 55%). The overall evaluation of the two operators showed that Sensor probe was significantly more accurate in detecting subgingival calculi in the middle (63% vs. 53%) and the apical thirds of the root site (75% vs. 52%). The accuracy of calculus detection for all sites combined with Sensor probe showed 64% accuracy, which was significantly better than the 54% accuracy of the conventional probe.

## Discussion

In this study, we developed a Sensor probe using a 0.5 mm diameter optical fiber and a 635 nm excitation semiconductor laser that could detect calculus with a diameter of 300 μm at a distance of 100 μm from the optical fiber end when it is cut perpendicular to the optical axis (C-type), highlighting the usefulness of this device for detecting subgingival calculus. This result further support the previous finding that a 635 nm excitation light is useful enough to detect calculi [25].

We experimentally verified the change in the calculus detection performance owing to the shape of the end of the optical fiber. The results indicated that the highest calculus detection performance was achieved when the optical fiber was cut at an angle of 45° to the optical axis (B-type) and when the optical fiber end was placed at an angle of 10° or 20° to the tooth surface. In the B type, the light emitted from the cut surface was biased towards the sides, and the light intensity was strong locally, resulting in high calculus detection performance. Therefore, the end of the optical fiber must face a certain direction of calculus to achieve the highest performance, which presents a challenge to its clinical use. We decided to use a B-type optical fiber that exhibited the best performance for calculus detection. Considering these directional limitations, a rotation mechanism was attached to Sensor probe tip to change the optimal direction of the fiber end. As shown in Fig 1B, Sensor probe is configured with a mechanism (indicated in red) that directs the fiber in the proper direction. By this, it is possible to debride the periodontal pocket and correct the direction of the fiber end facing to the root surface, making the most of utilizing plastic fibers to be safely inserted into the periodontal pocket. Experimental results using periodontal pocket dentition models showed significant differences among operators. In terms of performance and specificity, the use of the Sensor probe tended to result in better specificity than conventional periodontal probes. Sensor probes can obtain both calculus fluorescence and hand sensory information during root surface probing, which may enable a more accurate understanding of the periodontal pocket.

Several studies have reported the results of calculus detection in periodontal pockets. Shakibaie *et al*. used Diagnodent^®^ [30–33] or DetecTar [34, 35] and conventional periodontal probes to study the effectiveness of calculus detection in typodonts. The results showed that the sensitivity and specificity of calculus detection varied depending on the skill of the operators but were improved by the use of calculus detection devices. The sensitivities of calculus detection by using conventional periodontal probes were reported at 49–60%, specificity at 65–73% and accuracy at 57–67% [33, 35]. Thus, the results of our study were 49%, 56%, and 54%, respectively. Conversely, the sensitivity of Sensor probe compared to the reported Diagnodent^®^ Pen and DetecTar^®^ was 41% vs. 67–77%, specificity was 77% vs. 75–94%, and accuracy was 64% vs. 77–81%, respectively. The low sensitivity of Sensor probe used in this study was particularly problematic due to the high number of false negatives. We defined the calculus-positive site by direct observation with the naked eye, and the site that contained a small amount of calculus was misdiagnosed as negative by Sensor probe because the fiber end did not pass over the calculus-attached area and the calculus appeared to be flat and small when viewed from a small angle of the fiber end. In this experiment, the root surface scanning method was not specified, and the operators were allowed to scan the root surfaces freely. Developing a method or acquiring a skill to scan the root surface, by utilizing 0.5 mm size fiber end, without any gaps may result in reduction of false negatives in the future. However, the high specificity of Sensor probe means that it can more accurately identify areas where calculi have been removed and areas where no calculi were originally present. This is expected to avoid unnecessary scaling and contribute the most to a reduction in periodontal tissue invasion.

In this study, we did not directly compare other calculus detection systems with Sensor probe because the Sensor probe was designed in a similar shape to the periodontal probe, especially in handling, to enable calculus detection in deep periodontal pockets that other instruments cannot reach, and the range of root surface probing, especially in molars, was greatly expanded. The thin plastic fiber (0.5 mm) used in Sensor probe results in more flexible end that makes them suitable for use in narrow, deep periodontal pockets compared to other detection system with solid end. Furthermore, Sensor probe detected calculi in deeper periodontal pockets significantly more accurately than the conventional periodontal probe. These results suggest that the application of the Sensor probe can be clinically effective for treating more advanced periodontal diseases with less invasive periodontal tissues, especially when the periodontal pockets are deep.

Considering its clinical application, the size of the device itself is also an important issue; therefore, it was designed to be approximately the size of two paperback books stacked on top of each other (14.7 x 10.0 x 4.5 cm). Another issue is that the rotation mechanism at the tip renders the probe less operable. This mechanism also slightly thickens the probe tip, thereby compromising the thinness of the single-mode optical fiber. However, single-mode plastic fibers are unbreakable, durable, and safe in various clinical situations. In addition, the plastic fiber end can be easily cut to expose fresh fiber and prevent hospital-acquired infections. The disadvantage of single-mode optical fibers is increased optical noise, which is addressed using a high-pass filter. This type of hard-duty use is not possible with fragile quartz fibers, which expands the possibilities of clinical applications.

Further prospects for this device include integration with an ultrasonic scaler for seamless calculus detection and removal. The development of such device that can detect and remove calculus can decrease chair-side time, may lead to efficient scaling and avoid overzealous instrumentation. Further research is required to address these issues.

At this point, there are inherent limitations to our study. Although the capability of Sensor probe consisted of a 635 nm-wavelength semiconductor laser and a 0.5 mm-diameter single-mode optical fiber to detect subgingival calculus with improved accuracy was noted, these results are obtained from experiments in vitro. In clinical situations, in vivo, presence of fluids adjacent to calculus such as blood, gingival crevicular fluid and saliva may affect the beneficial effect of Sensor probe. In addition, bacterial cells or blood clots may mask the detection capability. In the present study, existence of water did not affect the effect of Sensor probe (data not shown), but further study would be needed to address this issue. Furthermore, in the current, in vitro, study, calculus attachment sites were defined as those easily visible to the naked eye under bright light. In the clinical setting, subgingival calculus deposits are not externally visible, this may have biased the results of the accuracy comparison between the methods. Moreover, in the current study, calibration between the two operators who compared the detection capability of Sensor probe, and a conventional periodontal probe was not performed. Both operators practiced the operation of the Sensor probe for only 5 min using an extracted tooth. There was a small variation between the operators, with operator 1 showed significantly improve of accuracy by the use of Sensor probe at both middle and apical regions compared to that only in apical region for operator 2. The size of the calculus; diameter of 300 μm which can be detectable and the distance from the optical fiber end (100 μm) is another limiting factor. However, if one can detect the calculus in such size at the bottom of the periodontal pocket, it may lead to better root surface debridement.

## Conclusion

This in vitro study revealed the possibility of a new dental calculus detector, namely a Sensor probe, using a semiconductor laser with a wavelength of 635 nm for subgingival calculus detection. By validating the changes in calculus detection performance among several shapes of the optical fiber end, we found that the most sensitive calculus detection could be achieved using an optical fiber cut at an angle of 45° to the optical axis for clinical applications. In addition, the use of this Sensor probe improved the accuracy of calculi detection in deep periodontal pockets compared to a regular periodontal probe, in vitro. Future in vitro as well as real-world clinical trials are necessary to fully validate whether Sensor probe would perform well in clinical settings.

## Supporting information

S1 Data (XLSX)
